# Social determinants disadvantage score and liver health in the All of Us Research Program

**DOI:** 10.1007/s10654-025-01358-y

**Published:** 2026-01-24

**Authors:** Xinyuan Zhang, Longgang Zhao, Kai Zhang, David Vlahov, Yun Chen, Ann Hsing, Mindie H. Nguyen, Katherine A. McGlynn, Tamar Taddei, Lifang Hou, Xuehong Zhang

**Affiliations:** 1 Channing Division of Network Medicine, Brigham and Women’s Hospital and Harvard Medical School, Boston, MA USA; 2 Yale University School of Nursing, Orange, CT USA; 3 Department of Population and Community Health, College of Public Health, The University of North Texas Health Science Center at Fort Worth, Fort Worth, TX USA; 4 Yale University School of Public Health, New Haven, CT USA; 5 Department of Medicine, Stanford Prevention Research Center and Department of Epidemiology and Population Health, Stanford University Medical Centre, Palo Alto, CA USA; 6 Division of Cancer Epidemiology and Genetics, National Cancer Institute, Bethesda, MD USA; 7 Department of Internal Medicine, Section of Digestive Diseases, Yale University School of Medicine, New Haven, CT USA; 8 VA Connecticut Health Care System, West Haven, CT USA; 9 Center for Population Epigenetics, Robert H. Lurie Comprehensive Cancer Center and Department of Preventive Medicine, Northwestern University Feinberg School of Medicine, Suite 1400, 680 N Lake Shore Drive, Chicago Illinois, 60611 USA; 10 Department of Nutrition, Harvard T.H. Chan School of Public Health, Boston, MA USA

**Keywords:** Social determinants of health, Steatotic liver disease, Liver cirrhosis, Hepatocellular carcinoma, Chronic hepatitis

## Abstract

Social determinants of health (SDOH) are crucial in shaping liver health outcomes, yet comprehensive assessments that span key SDOH domains are lacking. To address this knowledge gap, we developed a Social Determinants Disadvantage Score (SDDS) and examined its association with major adverse liver conditions. We conducted a cross-sectional analysis of 117,783 participants from the All of Us Research Program. The SDDS was systematically constructed using validated questionnaires covering economic stability, education, healthcare access and quality, neighborhood and built environment, and social and community context. Each question was scored on a 0 (most advantage) to 1 (most disadvantage) scale. Total SDDS was calculated as the mean of all questions, ranging from 0 to 1. We used logistic regression models to estimate odds ratios (ORs) and 95% confidence intervals (CIs) for the associations of SDDS with total and individual adverse liver conditions, including steatotic liver disease (SLD), metabolic dysfunction-associated steatohepatitis (MASH), alcoholic liver disease (ALD), cirrhosis, hepatocellular carcinoma (HCC), chronic hepatitis B virus (HBV), chronic hepatitis C virus (HCV), and hepatic failure based on the Electronic Health Record. Higher SDDS was associated with a higher risk of adverse liver conditions. The highest SDDS quintile (most disadvantaged) compared to the lowest SDDS quintile had an OR = 1.67 (95% CI: 1.55–1.79) for total adverse liver condition risk after adjusting for age, sex, race, and other covariates. Similar associations were observed for individual liver conditions. Per 10% higher SDDS, the adjusted OR (95% CI) was 1.25 (1.22–1.29) for SLD, 1.27 (1.19–1.35) for MASH, 1.15 (0.99–1.34) for ALD, 1.31 (1.25–1.39) for cirrhosis, 1.35 (1.15–1.59) for HCC, 1.24 (1.14–1.35) for HBV infection, 1.40 (1.33–1.48) for HCV infection, and 1.35 (1.21–1.50) for hepatic failure. Consistent associations were found for disadvantages in individual SDOH domains, score excluding missingness, and score with selected factors. The SDDS provides a comprehensive, validated tool for assessing SDOH and their associations with liver health. Our findings highlight significant associations between social disadvantage and the prevalence of adverse liver conditions, emphasizing the need for future longitudinal studies to inform targeted interventions.

## Introduction

Social determinants of health (SDOH) are the conditions in the environments where people are born, live, learn, work, play, and age. According to the recent U.S. Department of Health and Human Services (HHS) framework, SDOH can be grouped into five domains: economic stability, education, health care access and quality, neighborhood and built environment, and social and community context [1]. They play vital roles in human health, including liver health [2].

The burden of adverse liver conditions is large and increasing [3]. Worldwide, the prevalence of steatotic liver disease (SLD) is estimated at 30.1% [4], and in the U.S. National Health and Nutrition Examination Survey (NHANES), the prevalence is estimated to be 34.2% [5]. Approximately 2 million deaths worldwide annually are attributable to liver disease: 1 million due to cirrhosis and 1 million due to hepatitis B or C virus (HBV/HCV), and hepatocellular carcinoma (HCC) [6]. A few SDOH variables alone or in combination have been examined for their associations with adverse liver conditions. For example, higher prevalences of low income and food insecurity have been observed in SLD [7, 8]. Additionally, the presence of poverty has been found to be associated with not only a higher prevalence of various chronic liver diseases but also with higher mortality among those with liver disease [9, 10]. These studies laid the foundation for our understanding of liver health SDOH and also pointed to knowledge gaps. First, studies to date have often focused on single or limited domains, lacking comprehensive alignment with the five key SDOH domains outlined by major frameworks. Second, many key SDOH variables, especially in the neighborhood and built environment domain and the social and community context domain, are not consistently collected using validated questionnaires in epidemiological studies. Third, data on the total and the diverse spectrum of adverse liver conditions are lacking. Last, a widely used quantitative measure encompassing multiple SDOH domains is lacking in clinical practice [11], making it challenging for physicians to effectively incorporate SDOH into patient care. There is also a lack of rigorous, validated composite indices for SDOH in population studies [12].

Our overarching goal is to advance, support, and promote equity in liver health, focusing on SDOH domains that represent an unmet need of national and global relevance. We used data from the All of Us Research Program, a large and diverse sample of the U.S. population, to address the aforementioned key knowledge gaps. The first aim of this study was to develop a valid, easy-to-use, easy-to-translate Social Determinants Disadvantage Score (SDDS) to describe structural. The second aim of this study was to address urgent knowledge gaps in structural SDOH and liver health. We hypothesized that more disadvantages in SDOH, i.e., a higher SDDS, would be associated with a higher prevalence of adverse liver conditions.

## Methods

### Study population

This is a cross-sectional study of the All of Us Research Program, a national ongoing prospective cohort aimed at advancing precision medicine. A detailed description of the design and enrollment process has been described elsewhere [13]. Eligible participants are individuals aged ≥ 18 years residing in the U.S. who provide consent for access to their electronic health records, surveys, and optionally contribute biospecimens and physical measurements. The All of Us Institutional Review Board determined activities utilizing the Controlled Tier do not constitute research involving human subjects.

### Definition of SDOH

**Fig. 1:**
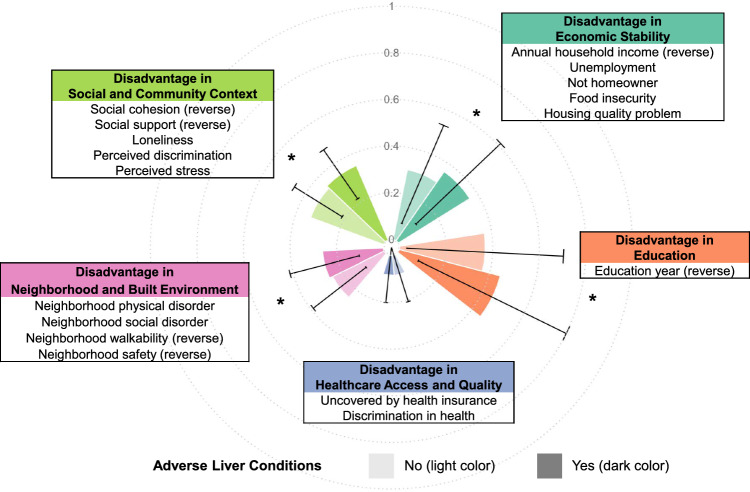
Social Determinants Disadvantage Score (SDDS) structural components and mean scores of each domain by adverse liver conditions. Each domain score takes the mean of all variables in the domain, ranging from 0 (most advantage) to 1 (most disadvantage). Higher scores indicate higher disadvantages. Total SDDS takes the mean of all domain scores, ranging from 0 (most advantage) to 1 (most disadvantage). Adverse liver conditions include any of the following conditions: steatotic liver disease, metabolic dysfunction-associated steatohepatitis, alcoholic liver disease, cirrhosis, hepatocellular carcinoma, chronic hepatitis B virus infection, chronic hepatitis C virus infection, and hepatic failure. * indicates *t*-test *P* < 0.001 comparing individuals with or without adverse liver conditions

Controlled Tier dataset version 7 was analyzed in this study. From November 1, 2021 to June 30, 2022, 29% (N = 117,783) of eligible All of Us participants completed the SDOH survey and other basic surveys containing topics related to SDOH were included for analysis. The SDOH survey utilized validated questionnaires and demonstrated good to excellent reliability [14, 15]. Survey questions were scaled as categorical variables or scored as continuous variables in alignment with the literature [14]. Scales and scores were framed into five domains of SDOH according to the HHS framework: economic stability, education, health care access and quality, neighborhood and built environment, and social and community context. Each domain consists of one to five scales or scores, as specified in Fig. 1.

### Developing the SDDS in the All of Us

We followed a systematic two-step approach to quantify the structural SDOH. In the first step, according to the original scale of the questionnaire-based SDOH variables, we standardized the directionality so that higher scores consistently reflect greater disadvantages. Categorical variables were coded such that the most advantaged category was assigned a score of 0, while the most disadvantaged category received a score of 1. Continuous variables’ scores were standardized to the range from 0–1. In the second step, each domain score was calculated as the mean score of variables in the domain. The final SDDS was calculated as the mean of all variables, resulting in a score ranging from 0 to 1, with higher scores indicating greater disadvantage. We used two approaches to handle missing variables: (1) calculating the mean score regardless of missing values and (2) setting the SDDS score to missing if any variable was missing. We compared these methods to assess the impact of missing data on the reliability and fit of the SDDS score.

### Definition of adverse liver conditions

The All of Us Research Program employs Observational Medical Outcomes Partnership (OMOP) Common Data Model Version 5 infrastructure to ensure feasibility and standardization across electronic health record (EHR) [16, 17]. Medical condition diagnoses from the EHR encompass all cases and were harmonized by definitions.

We defined adverse liver events by the presence of major individual chronic liver diseases including SLD, metabolic dysfunction-associated steatohepatitis (MASH), alcoholic liver disease (ALD), chronic HBV infection, chronic HCV infection, and/or their complications including liver cirrhosis, HCC, and hepatic failure [13]. A list of All of Us conditions matching each disease category is presented in Supplementary Table S1. These conditions are not mutually exclusive; individuals with multiple conditions are counted in all applicable categories. For example, a participant coded with both SLD and MASH is included in both categories; a participant coded with chronic HBV infection and HCC is included in both categories.

### Covariates

Information on basic personal demographics and key lifestyle behaviors was collected from surveys including age, sex at birth, race, ethnicity, birthplace, active duty, marital status, smoking status (lifetime cigarettes), and alcohol intake (drink frequency in the past year) [13, 16, 17]. Comorbidities including obesity and type 2 diabetes mellitus (T2DM) were identified from the EHR medical condition diagnoses.

### Statistical analysis

We used logistic regression models to estimate the odds ratio (OR) and 95% confidence interval (CI) of the risk of adverse liver conditions, total and individual, associated with SDOH, as assessed by the total SDDS and individual domain disadvantage level. Mode 1 is unadjusted. Model 2 is adjusted for age, sex, race, ethnicity, birthplace, marital status, smoking status, and alcohol intake. Model 3 is further adjusted for obesity and T2DM. In secondary analyses, we examined each individual SDOH variable and individual liver disease conditions. We used factor analysis for variable reduction, following similar steps of a validated Neighborhood Deprivation Index. [18] Variables with a loading score > 0.4 and tested for Cronbach's alpha correlation coefficient among the factors > 0.7 were selected and averaged as the alternative SDDS. We stratified the analyses by age group (≥ 65 or < 65 years old) and sex. A two-tailed p-value < 0.05 was used for statistical significance. All statistics were performed in the All of Us Researcher Workbench R version 4.3.3.

## Results

Table 1 shows the characteristics of the 117,783 participants according to SDDS quintile categories. We found there was an inverse association between age and SDDS, while the proportion of Black or African American individuals, Hispanic or Latino ethnicity, and females increases as the disadvantage increases. Supplementary Table S2 shows the characteristics of the group with at least one SDOH variable missing. This group is more similar to the high-disadvantage quintile categories.

**Table 1:** Characteristics of the study population according to the Social Determinants Disadvantage Score (SDDS) by quintile categories

	Quintile of social determinants disadvantage score				
	Q1 (most advantage)	Q2	Q3	Q4	Q5 (most disadvantage)
N	23,557	23,557	23,557	23,556	23,556
SDDS (mean (SD))	0.14 (0.03)	0.21 (0.02)	0.26 (0.02)	0.33 (0.02)	0.47 (0.08)
Age (mean (SD))	58.10 (13.92)	60.42 (15.51)	60.28 (16.74)	57.56 (17.92)	51.93 (16.79)
*Race (N (%))*					
Asian	760 (3.2)	617 (2.6)	645 (2.7)	668 (2.8)	491 (2.1)
Black or African American	601 (2.6)	888 (3.8)	1260 (5.3)	2046 (8.7)	4369 (18.5)
Middle Eastern or North African	111 (0.5)	88 (0.4)	99 (0.4)	98 (0.4)	102 (0.4)
More than one population	327 (1.4)	363 (1.5)	378 (1.6)	451 (1.9)	572 (2.4)
White	19,766 (83.9)	19,654 (83.4)	19,100 (81.1)	17,601 (74.7)	13,611 (57.8)
Other or unavailable	1992 (8.5)	1947 (8.3)	2075 (8.8)	2692 (11.4)	4411 (18.7)
*Ethnicity (N (%))*					
Not Hispanic or Latino	21,199 (90.0)	21,262 (90.3)	21,105 (89.6)	20,381 (86.5)	18,448 (78.3)
Hispanic or Latino	1171 (5.0)	1298 (5.5)	1565 (6.6)	2263 (9.6)	4020 (17.1)
Other or unavailable	1187 (5.0)	997 (4.2)	887 (3.8)	912 (3.9)	1088 (4.6)
*Sex at birth (N (%))*					
Male	9187 (39.0)	8743 (37.1)	8250 (35.0)	7595 (32.2)	6579 (27.9)
Female	13,529 (57.4)	14,211 (60.3)	14,814 (62.9)	15,446 (65.6)	16,451 (69.8)
Other or unavailable	841 (3.6)	603 (2.6)	493 (2.1)	515 (2.2)	526 (2.2)
*Birthplace (N (%))*					
USA	20,442 (86.8)	21,019 (89.2)	21,047 (89.3)	20,833 (88.4)	20,207 (85.8)
Other	2275 (9.7)	1954 (8.3)	2033 (8.6)	2267 (9.6)	2923 (12.4)
Unavailable	840 (3.6)	584 (2.5)	477 (2.0)	456 (1.9)	426 (1.8)
*Marital status (N (%))*					
Married	18,595 (78.9)	16,187 (68.7)	13,651 (57.9)	10,266 (43.6)	5829 (24.7)
Living with partner	887 (3.8)	1108 (4.7)	1301 (5.5)	1521 (6.5)	1896 (8.0)
Never married	1160 (4.9)	2239 (9.5)	3477 (14.8)	5187 (22.0)	7323 (31.1)
Divorced	1403 (6.0)	2130 (9.0)	2799 (11.9)	3855 (16.4)	5169 (21.9)
Separated	107 (0.5)	158 (0.7)	241 (1.0)	385 (1.6)	993 (4.2)
Widowed	539 (2.3)	1100 (4.7)	1543 (6.6)	1739 (7.4)	1534 (6.5)
Unavailable	866 (3.7)	635 (2.7)	545 (2.3)	603 (2.6)	812 (3.4)
*Smoking: 100 Cigs Lifetime (N (%))*					
No	16,821 (71.4)	15,343 (65.1)	14,482 (61.5)	13,900 (59.0)	12,363 (52.5)
Yes	6397 (27.2)	7900 (33.5)	8652 (36.7)	9251 (39.3)	10,651 (45.2)
Unavailable	339 (1.4)	314 (1.3)	423 (1.8)	405 (1.7)	542 (2.3)
*Alcohol: Drink Frequency Past Year (N (%))*					
Never	2268 (9.6)	2777 (11.8)	3444 (14.6)	4112 (17.5)	5202 (22.1)
Monthly or less	5099 (21.6)	6042 (25.6)	6628 (28.1)	7547 (32.0)	8586 (36.4)
2 to 4 per month	5401 (22.9)	5133 (21.8)	4956 (21.0)	4562 (19.4)	3671 (15.6)
2 to 3 per week	4810 (20.4)	4181 (17.7)	3642 (15.5)	3075 (13.1)	1945 (8.3)
4 or more per week	5178 (22.0)	4566 (19.4)	3813 (16.2)	2812 (11.9)	1568 (6.7)
Unavailable	801 (3.4)	858 (3.6)	1074 (4.6)	1448 (6.1)	2584 (11.0)
Type 2 diabetes mellitus, (N (%))	1425 (6.0)	1975 (8.4)	2487 (10.6)	2890 (12.3)	3873 (16.4)
Obesity, (N (%))	2590 (11.0)	3330 (14.1)	3899 (16.6)	4576 (19.4)	5776 (24.5)
*Annual household income (N (%))*					
Less than 25 k	53 (0.3)	337 (1.6)	1042 (5.0)	3221 (15.5)	10,220 (50.1)
25 k—50 k	431 (2.1)	1674 (8.1)	3732 (17.9)	6084 (29.4)	5906 (28.9)
50 k—75 k	1162 (5.6)	3290 (15.9)	4717 (22.7)	4922 (23.8)	2548 (12.5)
75 k—100 k	2178 (10.5)	3984 (19.2)	4145 (19.9)	3040 (14.7)	977 (4.8)
100 k—150 k	5400 (25.9)	5980 (28.8)	4345 (20.9)	2362 (11.4)	568 (2.8)
150 k—200 k	4072 (19.5)	2705 (13.0)	1491 (7.2)	609 (2.9)	113 (0.6)
More than 200 k	7539 (36.2)	2780 (13.4)	1350 (6.5)	478 (2.3)	75 (0.4)
Employed for wages (N (%))	3362 (14.3)	8951 (38.0)	11,411 (48.4)	11,895 (50.5)	12,933 (54.9)
*Current home own (N (%))*					
Own	281 (1.2)	787 (3.3)	1161 (4.9)	1900 (8.1)	2972 (12.6)
Rent	21,765 (92.4)	19,797 (84.0)	17,345 (73.6)	13,065 (55.5)	5646 (24.0)
Other arrangement	549 (2.3)	2222 (9.4)	4386 (18.6)	7820 (33.2)	13,996 (59.4)
Unavailable	962 (4.1)	751 (3.2)	665 (2.8)	771 (3.3)	942 (4.0)
Food insecurity (N (%))	24 (0.1)	135 (0.6)	591 (2.5)	2460 (10.4)	12,532 (53.2)
Housing quality problem (N (%))	405 (1.8)	1540 (6.9)	3085 (13.9)	6017 (27.2)	12,509 (55.7)
*Education year (N (%))*					
Under Twelve Or GED	253 (1.1)	879 (3.8)	1725 (7.5)	3391 (14.8)	7031 (30.7)
College One to Three	1704 (7.5)	3754 (16.4)	5675 (24.7)	7351 (32.0)	8962 (39.1)
College Graduate	6594 (29.2)	7704 (33.7)	7873 (34.3)	7021 (30.6)	4722 (20.6)
Advanced Degree	14,038 (62.1)	10,540 (46.1)	7711 (33.5)	5198 (22.6)	2205 (9.6)
Covered by health insurance (N (%))	22,692 (99.9)	22,848 (99.7)	22,808 (99.1)	22,290 (97.3)	20,608 (90.9)
Discrimination in health (mean (SD))	0.07 (0.10)	0.10 (0.13)	0.13 (0.14)	0.17 (0.16)	0.25 (0.19)
Neighborhood physical disorder (mean (SD))	0.09 (0.12)	0.14 (0.15)	0.19 (0.16)	0.25 (0.18)	0.36 (0.20)
Neighborhood social disorder (mean (SD))	0.09 (0.10)	0.14 (0.13)	0.19 (0.14)	0.25 (0.16)	0.38 (0.19)
Neighborhood walkability (mean (SD))	3.06 (0.81)	2.81 (0.89)	2.70 (0.92)	2.67 (0.93)	2.68 (0.88)
Neighborhood safety (mean (SD))	3.88 (0.29)	3.76 (0.42)	3.64 (0.53)	3.42 (0.68)	2.89 (0.89)
Social cohesion (mean (SD))	4.27 (0.56)	4.02 (0.59)	3.86 (0.60)	3.64 (0.63)	3.21 (0.77)
Social support (mean (SD))	4.55 (0.59)	4.22 (0.79)	3.94 (0.90)	3.61 (1.01)	3.06 (1.11)
Loneliness (mean (SD))	0.35 (0.12)	0.40 (0.13)	0.44 (0.14)	0.49 (0.16)	0.57 (0.17)
Perceived discrimination (mean (SD))	0.08 (0.10)	0.11 (0.11)	0.14 (0.13)	0.18 (0.15)	0.30 (0.22)
Perceived stress (mean (SD))	0.47 (0.10)	0.48 (0.09)	0.49 (0.10)	0.50 (0.11)	0.54 (0.12)

Of the 117,783 participants, 10,796 (9.17%) had at least one prevalent adverse liver condition in the EHR. Supplementary Table S3 shows characteristics according to the adverse liver condition. Figure 1 shows the mean scores of each domain by adverse liver conditions. Overall, the disadvantage in SDOH was associated with a higher prevalence of adverse liver conditions (Table 2). Specifically, compared to individuals in the lowest SDDS quintile (mean ± SD score = 0.14 ± 0.03; most advantaged, reference group), those in the highest SDDS quintile (mean ± SD score = 0.47 ± 0.08) had an OR of 1.67 (95% CI: 1.55, 1.79), after adjusting for age, sex, race, ethnicity, birthplace, marital status, smoking status, and alcohol intake (Model 2). Per 10% increase in the SDDS, the adjusted OR (95% CI) was 1.17 (1.15, 1.19) in Model 2 and 1.09 (1.07, 1.11) in Model 3 after further adjusting for obesity and T2DM. Excluding individuals with missingness in any SDOH variable showed consistently that disadvantage was significantly associated with adverse liver conditions, and that missingness was also an indicator for a higher risk of having adverse liver conditions (adjusted OR comparing to reference quintile = 1.33 (95% CI: 1.23, 1.43); Table 2, Model 2). In Model 3 after further adjusting for obesity and T2DM, the OR (95% CI) per 10% higher disadvantage in the individual domain was 1.04 (1.03, 1.05) for economic stability, 1.03 (1.02, 1.03) for education, 0.99 (0.97, 1.01) for health care access and quality, 1.02 (1.00, 1.03) for neighborhood and build environment, and 1.05 (1.03, 1.07) for social and community context (Supplementary Table S4). The associations of SDDS with adverse liver conditions remained significant in subgroups stratified by age (< 65 years old, ≥ 65 years old; Supplementary Table S5) and by sex (female, male; Supplementary Table S6).

**Table 2:** Associations between Social Determinants Disadvantage Score (SDDS) and adverse liver conditions

	Total N	Condition N	Model 1	Model 2	Model 3
*SDDS*					
Quintile 1 (most advantage)	23,557	1630	1 (ref)	1 (ref)	1 (ref)
Quintile 2	23,557	1879	1.17 (1.09, 1.25)	1.05 (0.98, 1.13)	1.00 (0.93, 1.07)
Quintile 3	23,557	2074	1.30 (1.21, 1.39)	1.12 (1.04, 1.20)	1.00 (0.93, 1.08)
Quintile 4	23,556	2313	1.46 (1.37, 1.56)	1.26 (1.17, 1.35)	1.06 (0.99, 1.14)
Quintile 5 (most disadvantage)	23,556	2900	1.89 (1.77, 2.01)	1.67 (1.55, 1.79)	1.26 (1.17, 1.36)
Per 10% higher disadvantage			1.19 (1.17, 1.21)	1.17 (1.15, 1.19)	1.09 (1.07, 1.11)
*SDDS, with missing indicator*					
Quintile 1 (most advantage)	14,426	899	1 (ref)	1 (ref)	1 (ref)
Quintile 2	14,426	1046	1.18 (1.07, 1.29)	1.06 (0.96, 1.16)	1.00 (0.91, 1.10)
Quintile 3	14,426	1143	1.29 (1.18, 1.42)	1.11 (1.01, 1.21)	1.01 (0.92, 1.11)
Quintile 4	14,425	1240	1.42 (1.29, 1.55)	1.20 (1.09, 1.32)	1.03 (0.94, 1.14)
Quintile 5 (most disadvantage)	14,425	1619	1.90 (1.75, 2.07)	1.62 (1.48, 1.78)	1.30 (1.18, 1.43)
Missing	45,655	4849	1.79 (1.66, 1.92)	1.33 (1.23, 1.43)	1.19 (1.10, 1.29)
Per 10% higher disadvantage			1.20 (1.18, 1.23)	1.20 (1.17, 1.23)	1.11 (1.08, 1.14)

Odds ratio (OR) and 95% confidence interval (CI) are estimated from logistic regression model

Mode 1 is unadjusted. Model 2 is adjusted for age, sex, race, ethnicity, birthplace, marital status, smoking status, and alcohol intake. Model 3 is further adjusted for type 2 diabetes mellitus and obesity

For the top individual liver disease, we identified 4622 SLD, 718 MASH, 152 ALD, 424 chronic HBV infection, 1011 chronic HCV infection, 1024 liver cirrhosis, 110 HCC, and 243 hepatic failure cases. Table 3 showed a significant association between higher SDDS and prevalence of all conditions except ALD. The adjusted OR (95% CI) per unit increase of SDDS was 1.25 (1.22, 1.29) for SLD, 1.27 (1.19, 1.35) for MASH, 1.15 (0.99, 1.34) for ALD, 1.31 (1.25, 1.39) for cirrhosis, 1.35 (1.15, 1.59) for HCC, and 1.35 (1.21, 1.50) for hepatic failure in Model 2. Chronic HBV and HCV infections were also associated with higher SDDS, with ORs of 1.24 (95% CI: 1.14, 1.35) and 1.40 (95% CI: 1.33, 1.48), respectively. Further adjusting for obesity and T2DM attenuated the associations towards null, especially for ALD (Table 3, Model 3). For HCC as the outcome, further adjusting for chronic HBV and HCV infections attenuated the associations (OR = 1.24; 95% CI: 1.05, 1.47). The multivariable-adjusted associations of SDDS excluding missingness and liver conditions (Table 3) and individual SDOH domains (Supplementary Table S4) with individual liver diseases were overall consistent with the primary results. Taking these conditions together, 4661 had records of one condition, 1042 had 2 different conditions, and 463 had ≥ 3 different conditions. In the ordinal logistic regression, both SDDS and SDDS excluding missingness were significantly associated with more conditions (Supplementary Table S7).

**Table 3:** Associations between per 10% higher disadvantage in Social Determinants Disadvantage Score (SDDS) and individual major chronic liver diseases

Conditions	SDDS				SDDS, with missing indicator			
	Condition N	Model 1	Model 2	Model 3	Condition N	Model 1	Model 2	Model 3
Steatotic liver disease (SLD)	4622	1.26 (1.24, 1.29)	1.25 (1.22, 1.29)	1.12 (1.09, 1.15)	2615	1.28 (1.24, 1.32)	1.29 (1.24, 1.34)	1.14 (1.10, 1.19)
Metabolic dysfunction-associated steatohepatitis (MASH)	718	1.29 (1.22, 1.36)	1.27 (1.19, 1.35)	1.09 (1.02, 1.17)	398	1.29 (1.20, 1.39)	1.28 (1.17, 1.40)	1.08 (0.99, 1.19)
Alcoholic liver disease (ALD)	152	1.16 (1.03, 1.31)	1.15 (0.99, 1.34)	1.04 (0.90, 1.21)	80	1.13 (0.95, 1.35)	1.13 (0.90, 1.40)	1.01 (0.81, 1.26)
Liver cirrhosis	1024	1.40 (1.34, 1.46)	1.31 (1.25, 1.39)	1.21 (1.15, 1.28)	512	1.44 (1.36, 1.54)	1.38 (1.27, 1.49)	1.25 (1.15, 1.35)
Hepatocellular carcinoma (HCC)	110	1.36 (1.19, 1.55)	1.35 (1.15, 1.59)	1.27 (1.08, 1.50)	58	1.48 (1.24, 1.78)	1.57 (1.26, 1.97)	1.45 (1.15, 1.82)
Chronic hepatitis B virus infection	424	1.35 (1.27, 1.45)	1.24 (1.14, 1.35)	1.19 (1.09, 1.29)	214	1.34 (1.21, 1.48)	1.28 (1.14, 1.45)	1.22 (1.08, 1.38)
Chronic hepatitis C virus infection	1011	1.64 (1.57, 1.71)	1.40 (1.33, 1.48)	1.37 (1.30, 1.44)	513	1.67 (1.57, 1.77)	1.48 (1.37, 1.59)	1.43 (1.33, 1.54)
Hepatic failure	243	1.40 (1.28, 1.52)	1.35 (1.21, 1.50)	1.23 (1.10, 1.37)	119	1.50 (1.32, 1.70)	1.52 (1.31, 1.78)	1.37 (1.17, 1.60)

Odds ratio (OR) and 95% confidence interval (CI) are estimated from logistic regression model

Mode 1 is unadjusted. Model 2 is adjusted for age, sex, race, ethnicity, birthplace, marital status, smoking status, and alcohol intake. Model 3 is further adjusted for type 2 diabetes mellitus and obesity

Factor analysis and variable reduction selected 13 SDOH variables with at least one variable in each domain (Table 4). Each variable and the alternative score using these 13 variables showed significant, similar results that higher disadvantage was associated with higher prevalence of adverse liver conditions. Per 10% increase in the alternative SDDS, the adjusted OR (95% CI) was 1.16 (1.14, 1.18) in Model 2 and 1.08 (1.06, 1.10) in Model 3, comparable to the SDDS and SDDS excluding missingness.

**Table 4:** Associations of factor analysis selected individual social determinants and alternative Social Determinants Disadvantage Score (SDDS; mean of the selected variables) with total adverse liver conditions

	Total N	Condition N	Model 1	Model 2	Model 3
*Variables with loading* > *0.4 in any factor, per 10% higher disadvantage *^*a*^					
Annual household income (reverse)			1.07 (1.06, 1.08)	1.04 (1.03, 1.05)	1.01 (1.00, 1.02)
Not homeowner			1.01 (1.01, 1.02)	1.02 (1.02, 1.03)	1.01 (1.01, 1.02)
Education year (reverse)			1.07 (1.06, 1.08)	1.06 (1.05, 1.06)	1.03 (1.02, 1.03)
Discrimination in health			1.02 (1.01, 1.03)	1.04 (1.02, 1.05)	1.02 (1.01, 1.04)
Neighborhood physical disorder			1.03 (1.02, 1.05)	1.04 (1.03, 1.05)	1.02 (1.01, 1.04)
Neighborhood social disorder			1.05 (1.04, 1.06)	1.05 (1.04, 1.06)	1.03 (1.02, 1.04)
Neighborhood safety (reverse)			1.04 (1.04, 1.05)	1.03 (1.03, 1.04)	1.02 (1.01, 1.03)
Social cohesion (reverse)			1.03 (1.02, 1.04)	1.04 (1.02, 1.05)	1.02 (1.01, 1.03)
Social support (reverse)			1.05 (1.04, 1.06)	1.02 (1.01, 1.03)	1.01 (1.00, 1.02)
Loneliness			1.04 (1.03, 1.05)	1.04 (1.03, 1.06)	1.02 (1.01, 1.03)
Perceived discrimination			1.01 (1.00, 1.02)	1.06 (1.04, 1.07)	1.03 (1.02, 1.05)
Perceived stress			0.99 (0.97, 1.01)	1.06 (1.04, 1.08)	1.06 (1.04, 1.08)
*Alternative SDDS, mean of the above variables*					
Quintile 1 (most advantage)	23,553	1744	1 (ref)	1 (ref)	1 (ref)
Quintile 2	23,552	1926	1.11 (1.04, 1.19)	1.08 (1.01, 1.16)	1.00 (0.94, 1.08)
Quintile 3	23,552	2129	1.24 (1.16, 1.33)	1.21 (1.13, 1.30)	1.05 (0.98, 1.13)
Quintile 4	23,552	2216	1.30 (1.22, 1.39)	1.32 (1.23, 1.42)	1.07 (1.00, 1.15)
Quintile 5 (most disadvantage)	23,552	2779	1.67 (1.57, 1.78)	1.75 (1.62, 1.88)	1.30 (1.20, 1.41)
Missing	22	2	Not estimated	Not estimated	Not estimated
Per 10% higher disadvantage			1.14 (1.12, 1.15)	1.16 (1.14, 1.18)	1.08 (1.06, 1.10)

Odds ratio (OR) and 95% confidence interval (CI) are estimated from logistic regression model

^a^ Excluded any missing data in each variable

Mode 1 is unadjusted. Model 2 is adjusted for age, sex, race, ethnicity, birthplace, marital status, smoking status, and alcohol intake. Model 3 is further adjusted for type 2 diabetes mellitus and obesity

## Discussion

In this large-scale cross-sectional study, we proposed a systematically constructed score, SDDS, that captured all domains of disadvantage contributing to inequitable health outcomes. We found that disadvantage in structured SDOH, as assessed by individual domains, variables, and SDDS, was associated with poorer liver health. Disadvantage in economic stability, education, health care access and quality, neighborhood and built environment, and social and community context was independently associated with a higher likelihood of having total and individual adverse liver conditions. These associations remained robust after adjusting for key personal demographics and lifestyles such as age, sex, race, ethnicity, country of birth, marital status, smoking, alcohol drinking, obesity and T2DM. The associations remained consistent when excluding any missing data and using an alternative score with all five domains but fewer variables. The effect sizes illustrate that social disadvantage operates as a risk factor, comparable in magnitude to established metabolic and lifestyle factors. A 10% increase in the SDDS represents a meaningful shift in cumulative social disadvantage across multiple domains, and the corresponding increases in odds of adverse liver conditions underscore the clinical sensitivity of liver health to incremental social burden.

### In context with previous literature

Our study, for the first time and in a large sample size, reported the significant, independent associations of each SDOH domain with prevalent adverse liver conditions. Results are in line with previous literature on the associations of key individual variables on liver diseases. For example, a key assessment of economic stability, food insecurity, was associated with liver fibrosis and an increased risk of advanced fibrosis and cirrhosis in a cross-sectional study of 3502 US adults in NHANES [19]. However, for other domains, their associations with the prevalence or incidence of liver diseases in the general population have not been well quantified. Studies that simultaneously evaluate multiple SDOH domains are sparse.

A few studies have examined social factors in individuals with liver disease. A study of 11,107 participants with chronic liver disease in Italy found that low educational level was associated with higher disease severity [20]. In the U.S. national cohort of Veterans Health Administration (VA), rural residents had a lower likelihood of accessing gastroenterology or hepatology specialty care compared to their urban counterparts [21]. However, the comparison of quality of care with disease incidence or prognosis was out of the scope of these studies. A recent retrospective cohort study found that disadvantage in neighborhood-level SDOH was associated with mortality, incidence of adverse liver conditions, and incident cardiovascular disease in 15,904 individuals with SLD [22]. However, the neighborhood SDOH was assessed by only the average income, education, and occupation status of households in the neighborhood. For the social and community context, a study on liver transplant recipients found that social support was positively correlated with self-management and hope [23]. Taking together the more comprehensive results from our study, we hope to raise awareness of SDOH for liver health.

A recent study using the All of Us dataset developed an SDOH score specifically for coronary heart disease (CHD), showing that all five domains were associated with the prevalence of CHD [24]. Compared to their data-driven approach using elastic net regression, our method is independent of specific disease events, more resilient to data bias and missing data, and may have potential for generalizability across different diseases and studies. We plan to evaluate the SDDS across other conditions within the All of Us cohort and in external studies.

### Impact

Comprehensive individual-level data on SDOH are challenging to collect and may be prone to inaccuracies. We designed SDDS to be flexible and adaptable for use in diverse populations including study populations where data collection may be limited. For instance, if only one or two variables are available within a domain, these variables still should be able to effectively describe disadvantage status. By standardizing scores within the population, the SDDS is also adaptable to the heterogeneous distribution of data and the assessment tools used within each population. Compared to a few previous assessments of SDOH, our study captures wider domains and can examine their synergistic effects on health outcomes [25, 26]. Compared to other approaches to measuring SDOH using aggregate data, our methods individual-level variations [27–29]. Although future studies are warranted to validate this score and more data are needed to refine cutoff values, our conceptual approach to SDDS enables a standardized quantification and comparison of disadvantages, laying a foundation for consistent use in various settings. Because the SDDS provides a standardized, domain-based quantification of social disadvantage, it can be incorporated into screening. Clinically, the SDDS could be used with metabolic and lifestyle risk factors to flag patients whose social context places them at elevated risk for adverse liver outcomes. In public health practice, the SDDS can support population-level risk stratification, guide resource allocation, and inform upstream interventions that address modifiable social and structural drivers of liver disease.

### Strengths and limitations

This study has several notable strengths. It is the first to employ a structured SDOH framework for liver conditions, using one of the largest sample sizes available (> 100,000) by leveraging a nationwide representative study. The use of validated questionnaires and scales ensures the robustness and reliability of the data collected. Furthermore, our study includes diverse participants and considers groups underrepresented in biomedical research, such as diverse racial and ethnic groups. Importantly, we validated the SDDS against liver conditions, a critical and leading health concern, demonstrating the practical relevance and impact of this score on public health.

Limitations should be noted. First, although the All of Us reflects the diversity of the U.S., it cannot be described as a representative sample [13]. The results may not generalize to populations with different SDOH. Second, the All of Us program does not focus on any particular set of diseases, enabling the study of various diseases and health status but meanwhile limits the detailed, specific information on disease of interest, in this case, the diseases of the liver. Third, the potential underdiagnosis of liver disease by EHR highlights the need for future studies replicating our approaches. Despite this, our findings may still hold scientific validity, as the associations observed between social disadvantage and liver conditions are consistent with known risk patterns. Additionally, underdiagnosis could bias the results towards the null, suggesting that the true associations may be even stronger. Last, longitudinal follow-up studies are warranted to address the impact of SDOH on chronic, adverse liver conditions. Such studies would allow for causal inference, providing insights into how social disadvantage influences liver disease development and progression over time. They could also identify critical windows for intervention and inform strategies to mitigate the burden of liver disease in vulnerable populations.

To conclude, we developed the Social Determinants Disadvantage Score (SDDS) as a comprehensive tool for assessing social disadvantage in future studies. By validating this score against liver conditions, we addressed a significant knowledge gap in understanding the associations between SDOH and liver health. Our findings reveal clear and dose–response associations between higher SDDS and higher risk of adverse liver conditions, highlighting the importance of social factors in liver health and providing a strong foundation for future research and potential interventions.

## Data Availability

Data, data dictionary, and analysis script are available in the All of Us Researcher Workbench, a cloud-based platform where registered researchers can access Registered and Controlled Tier data.
